# Physiological responses and transcriptomic changes reveal the mechanisms underlying adaptation of *Stylosanthes guianensis* to phosphorus deficiency

**DOI:** 10.1186/s12870-021-03249-2

**Published:** 2021-10-13

**Authors:** Zhijian Chen, Jianling Song, Xinyong Li, Jacobo Arango, Juan Andres Cardoso, Idupulapati Rao, Rainer Schultze-Kraft, Michael Peters, Xiaohui Mo, Guodao Liu

**Affiliations:** 1 Institute of Tropical Crop Genetic Resources, Chinese Academy of Tropical Agricultural Sciences, Haikou, 571101 P.R. China; 2 Hainan Key Laboratory for Sustainable Utilization of Tropical Bioresources, College of Tropical Crops, Hainan University, Haikou, 570110 P.R. China; 3 Alliance of Bioversity International and International Center for Tropical Agriculture, Cali, A.A.6713 Colombia; 4 Root Biology Center, State Key Laboratory for Conservation and Utilization of Subtropical Agro-bioresources, College of Natural Resources and Environment, South China Agricultural University, Guangzhou, 510642 P.R. China

**Keywords:** *Stylosanthes*, P deficiency, Transcriptomics, P responsive genes, Root morphology, Pi homeostasis

## Abstract

**Background:**

Phosphorus (P) is an essential macronutrient for plant growth that participates in a series of biological processes. Thus, P deficiency limits crop growth and yield. Although *Stylosanthes guianensis* (stylo) is an important tropical legume that displays adaptation to low phosphate (Pi) availability, its adaptive mechanisms remain largely unknown.

**Results:**

In this study, differences in low-P stress tolerance were investigated using two stylo cultivars (‘RY2’ and ‘RY5’) that were grown in hydroponics. Results showed that cultivar RY2 was better adapted to Pi starvation than RY5, as reflected by lower values of relative decrease rates of growth parameters than RY5 at low-P stress, especially for the reduction of shoot and root dry weight. Furthermore, RY2 exhibited higher P acquisition efficiency than RY5 under the same P treatment, although P utilization efficiency was similar between the two cultivars. In addition, better root growth performance and higher leaf and root APase activities were observed with RY2 compared to RY5. Subsequent RNA-seq analysis revealed 8,348 genes that were differentially expressed under P deficient and sufficient conditions in RY2 roots, with many Pi starvation regulated genes associated with P metabolic process, protein modification process, transport and other metabolic processes. A group of differentially expressed genes (DEGs) involved in Pi uptake and Pi homeostasis were identified, such as genes encoding Pi transporter (PT), purple acid phosphatase (PAP), and multidrug and toxin extrusion (MATE). Furthermore, a variety of genes related to transcription factors and regulators involved in Pi signaling, including genes belonging to the PHOSPHATE STARVATION RESPONSE 1-like (PHR1), WRKY and the SYG1/PHO81/XPR1 (SPX) domain, were also regulated by P deficiency in stylo roots.

**Conclusions:**

This study reveals the possible mechanisms underlying the adaptation of stylo to P deficiency. The low-P tolerance in stylo is probably manifested through regulation of root growth, Pi acquisition and cellular Pi homeostasis as well as Pi signaling pathway. The identified genes involved in low-P tolerance can be potentially used to design the breeding strategy for developing P-efficient stylo cultivars to grow on acid soils in the tropics.

**Supplementary Information:**

The online version contains supplementary material available at 10.1186/s12870-021-03249-2.

## Background

Phosphorus (P) is one of the essential macronutrients for plant growth and development. P is involved in the processes of photosynthesis, respiration, energy metabolism and signal transduction in plants [1]. Furthermore, P is also an important structural component of various biomolecules in plant cells, including adenosine triphosphate (ATP), phospholipids, DNA and RNA [2]. Although total P is abundant in soils, P is easily immobilized by soil components into unavailable forms that cannot be directly utilized by plants [3]. Thus, low phosphate (Pi) availability is considered a major limiting factor for crop growth, especially in acid soils that occupy about 50% of the world’s arable land [3, 4]. To obtain high yields in traditional agricultural systems, farmers need to apply excessive quantities of P fertilizer, potentially leading to soil deterioration and eutrophication problems [5]. Furthermore, P fertilizer is derived from mined phosphate rock, which is a finite resource that is slowly depleting [6]. Therefore, improving the absorption and utilization of soil P can be an effective way for increasing crop yield and reducing fertilizer P application. Such improvements aim for the development of a more sustainable and environmentally sound agriculture.

To cope with low-P stress, plants have improved Pi uptake and homeostasis through a wide range of morphological, physiological and molecular changes, such as modifying root morphology and architecture, increasing secretion of organic acid and acid phosphatases, enhancing expression of high-affinity Pi transporters, developing symbioses with arbuscular mycorrhizal fungi, and regulating complex P signaling networks in plant cells [7, 8]. It has been well demonstrated that plants display plasticity in root growth under P deficiency by changing root morphology and architecture, and thus increasing acquisition of P from the soil [9, 10]. For example, increase in root length and root/shoot ratio is observed in maize (*Zea mays*) [11], faba bean (*Vicia faba*) [12], rapeseed (*Brassica napus*) and wheat (*Triticum aestivum*) [13] in response to low P supply. Furthermore, acid phosphatase activities are up-regulated by Pi deprivation in many plants, such as rice (*Oryza sativa*), soybean (*Glycine max*) and chickpea (*Cicer arietinum*), which could contribute to increased organic P utilization [14–16].

To date, a variety of P responsive genes have been identified to participate in Pi uptake and homeostasis [8, 17]. For example, PHOSPHATE STARVATION RESPONSE 1 (AtPHR1) in Arabidopsis and OsPHR2 in rice, encoding the MYB transcription factor, are the central regulators involved in Pi signaling pathway [8]. AtPHR1 is demonstrated to regulate a set of Pi starvation induced (PSI) genes through binding to the P1BS *cis* element of target genes [18]. Furthermore, a negative regulatory role for protein containing the SYG1/PHO81/XPR1 (SPX) domain in rice is documented where OsSPX1 suppresses the transcripts of several PSI genes, such as Pi transporters (*OsPT2* and *OsPT6*) and purple acid phosphatases (*OsPAP10*) [7]. A group of Pi transporters have been functionally characterized to be involved in Pi uptake and/or translocation in many plants; examples include: AtPT1 and AtPT2 from Arabidopsis [19, 20], OsPT1/9/10 from rice [21, 22] and GmPT5/7 from soybean [23, 24]. In addition, numerous purple acid phosphatase (PAP) homologues have also been demonstrated to function in Pi release from organic P, including AtPAP10/12/26 from Arabidopsis [25], OsPHY1 from rice [26], PvPAP1/3 from common bean (*Phaseolus vulgaris*) [27] and GmPAP14/33 from soybean [28, 29]. Furthermore, many other PSI genes have been identified to regulate root morphology and architecture, such as *expansin 2* (*GmEXPB2*) and *expansin 17* (*OsEXPA17*) [30, 31].

*Stylosanthes guianensis* (stylo) originates from the New World tropics, where acid soils are widely distributed [32]. Stylo is widely used in agricultural systems for livestock nutrition and soil improvement in South China [33, 34]. Owing to its greater ability to adapt to plant growth limiting factors in acid soils, such as P deficiency, aluminum (Al) and manganese (Mn) toxicity, stylo has been regarded as an important tropical legume for dissecting its adaptive mechanisms to nutrient-poor acid soil [35–37]. A P-efficient stylo genotype has been found to display the capacity to use organic P as well as tolerance to high Al through enhanced root APase activity and modified root growth response, respectively [37–39]. Although the studies mentioned above provide some insights into the mechanisms of low-P tolerance in stylo, little is known about those associated with differential gene expression patterns. Certainly, the overall changes in expression profile of PSI genes in stylo roots have not been documented, which can be potentially used as candidate genes for breeding stylo varieties with high level of tolerance to low P in acid soils. Accordingly, the effects of P deficiency on dry mass production, P concentration and APase activities of two stylo cultivars, ‘RY2’ and ‘RY5’, were analyzed in this study. Genome-wide transcriptomic analysis of stylo roots in response to P deficiency was further conducted using RNA-seq and the molecular mechanisms underlying adaptation of stylo to low-P stress are suggested.

## Results

### Characterization of low-P tolerance in two stylo cultivars

**Fig. 1:**
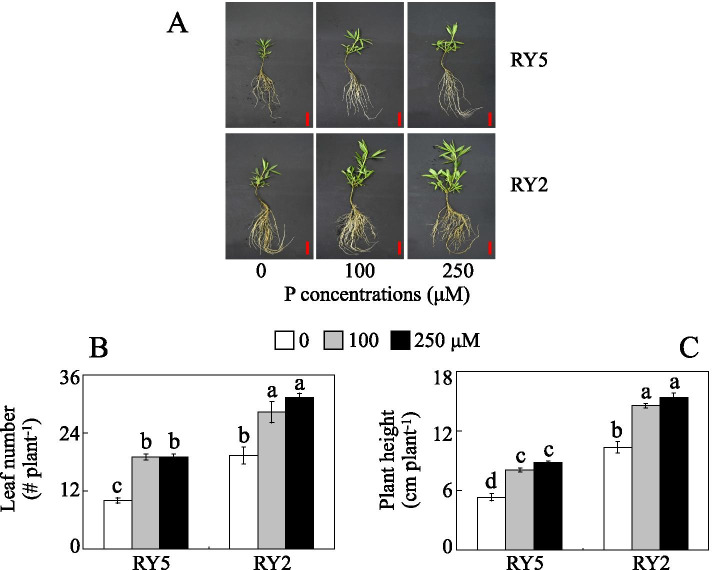
Stylo growth under different P levels. **a** Growth performance of stylo. **b** Plant height. **c** Leaf number. Fourteen-day-old stylo seedlings were subjected to 0, 100 and 250 μM KH_2_PO_4_ as low (Pi deprivation), moderate and high P supply treatments for 21 d, respectively. Each bar represents the mean of three independent replicates with standard error (SE). Different letters indicate significant differences among P treatments at *P*<0.05. Bar=5 cm

**Fig. 2:**
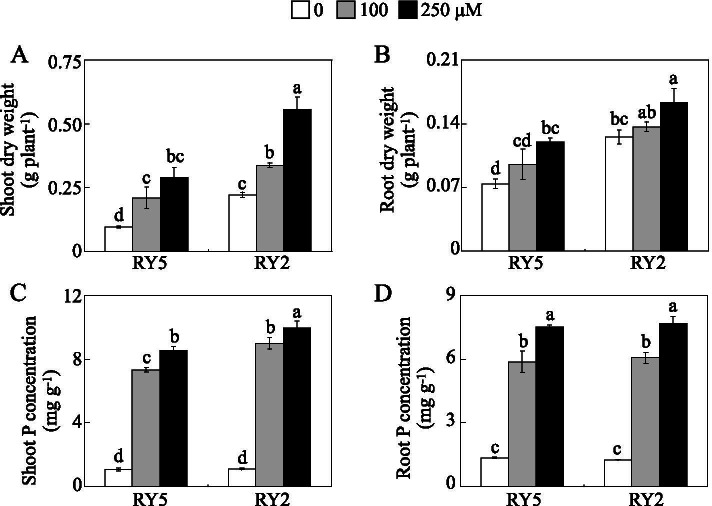
Plant dry weight and P concentration of stylo at different P treatments. **a** Shoot dry weight. **b** Root dry weight. **c** P concentration in shoot. **d** P concentration in root. Fourteen-day-old stylo seedlings were subjected to 0, 100 and 250 μM KH_2_PO_4_ as low (Pi deprivation), moderate and high P supply treatments for 21 d, respectively. Each bar represents the mean of three independent replicates with standard error (SE). Different letters indicate significant differences among P treatments at *P*<0.05

In this study, two stylo cultivars (‘RY2’ and ‘RY5’) were subjected to 0, 100 and 250 μM KH_2_PO_4_ as low (Pi starvation), moderate and high P supply treatments, respectively, for 21 d under hydroponic conditions. Results showed that both plant height and leaf number of the two cultivars tested were significantly affected by P treatments. Compared to the high P (250 μM P) treatment, low P availability resulted in decreased plant height and leaf number of both cultivars, especially under low P (0 μM P) treatment (Fig. 1). The values of relative decrease rates of plant height and leaf number were 39.5 and 47.4% in RY5, and were 32.8 and 38.5% in RY2 under 0 μM P treatment compared to those under high P (250 μM) application (Fig. 1b and c, Additional file 1). Similarly, compared to 250 μM P treatment, shoot and root dry weight were decreased by 38.4-66.5% in RY5 and 22.6-58.9% in RY2 with 0 μM P treatment (Fig. 2a and b). Furthermore, the relative decrease rates of shoot and root dry weight of RY5 were significantly higher than those of RY2 under 0 μM P condition (Additional file 1). Similar to the results of shoot and root dry weight, low P availability resulted in less P accumulation in shoots and roots of the two cultivars. P concentrations in shoots and roots were decreased by 82.0-87.5% in RY5 and 83.7-88.9% in RY2 with 0 μM P treatment, compared to those in 250 μM P treatment (Fig. 2c and d).

**Fig. 3:**
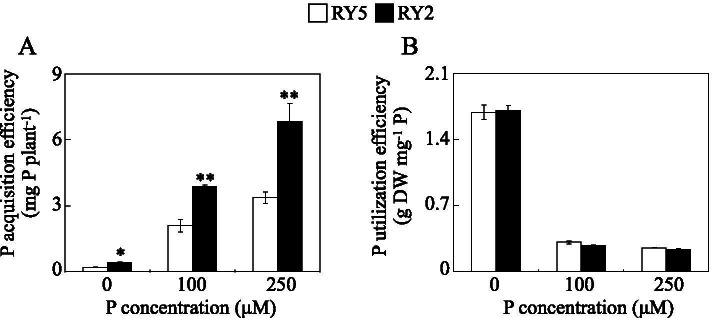
P acquisition and utilization efficiencies of stylo at different P levels. **a** P acquisition efficiency. **b** P utilization efficiency. Each bar represents the mean of three independent replicates with SE. Asterisks indicate significant differences between the two stylo cultivars. *, 0.01<*P*<0.05. **, 0.001<*P*<0.01

In addition, P acquisition and utilization efficiencies of the two cultivars evaluated were markedly influenced by P availability (Fig. 3). P acquisition efficiency of both cultivars was significantly increased by P supply in the solution. It was notable that P acquisition efficiency was higher in RY2 than in RY5 by more than 100.3, 84.7 and 102.7% under 0, 100 μM and 250 μM P treatments, respectively (Fig. 3a). In contrast, P utilization efficiency of the two cultivars was decreased by an increase in P supply, and P utilization efficiency of RY2 was similar to that of RY5 under the same P treatment (Fig. 3b). These results together show that stylo cultivar RY2 is more tolerant to low-P stress compared to RY5.

### Changes of root morphology and APase activities in response to Pi starvation

**Fig. 4:**
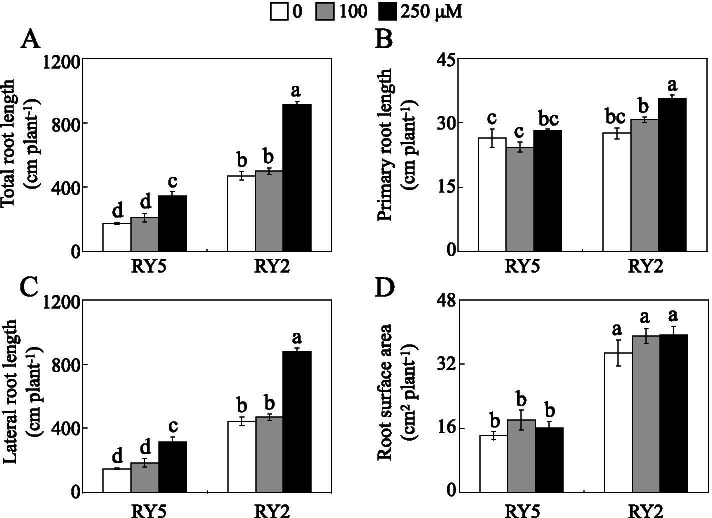
Stylo root growth at different P levels. **a** Total root length. **b** Primary root length. **c** Lateral root length. **d** Root surface area. Fourteen-day-old stylo seedlings were subjected to 0, 100 and 250 μM KH_2_PO_4_ as low (Pi deprivation), moderate and high P supply treatments for 21 d, respectively. Each bar represents the mean of three independent replicates with SE. Different letters indicate significant differences among P treatments at *P*<0.05

Root morphology was subsequently analyzed from the response of stylo to P deficiency. As shown in Fig. 4, both total root length and lateral root length declined in the two cultivars under 0 and 100 μM P treatments compared to high P application, but no significant differences were observed in the relative decrease rates of total root length and lateral root length between the two cultivars. Interestingly, total root length, lateral root length and root surface area in RY2 were 138.3-169.4, 152.9-198.9 and 116.6-144.8% higher than those in RY5 under 0 and 100 μM P treatments, respectively (Fig. 4).

**Fig. 5:**
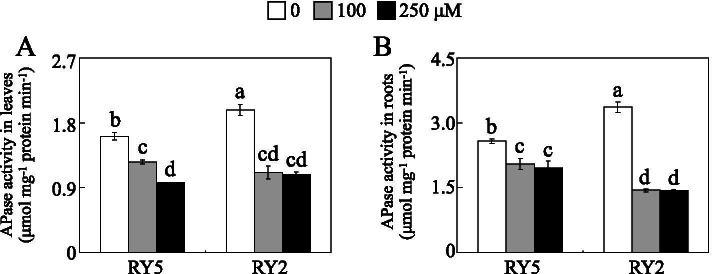
Determination of APase activities. **a** Leaf APase activity. **b** Root APase activity. Fourteen-day-old stylo seedlings were subjected to 0, 100 and 250 μM KH_2_PO_4_ as low (Pi deprivation), moderate and high P supply treatments for 21 d, respectively. Each bar represents the mean of three independent replicates with SE. Different letters indicate significant differences among P treatments at *P*<0.05

As increasing activity of APase is considered to be beneficial for plants in improving organic P utilization, APase activities in leaf and root of stylo under different P treatments were further assayed. Compared to 250 μM P, leaf and root APase activities were significantly increased by 0 μM P treatment in both stylo cultivars (Fig. 5). Leaf APase activities in RY5 and RY2 respectively increased by 66.9 and 83.3% under 0 μM P treatment compared to 250 μM P application. Root APase activities in RY5 and RY2 were 31.7 and 135.8% greater under 0 μM P treatment compared to 250 μM P application (Fig. 5). Furthermore, leaf and root APase activities in RY2 were about 22.8 and 30.3%, respectively, higher than those in RY5 under 0 μM P condition (Fig. 5).

### Transcriptomic changes in stylo roots in response to Pi starvation

To further investigate the global expression profile of Pi starvation responsive genes in stylo roots, transcriptomic analysis of the low-P tolerant stylo cultivar RY2 at 0 (LP) and 250 (HP) μM KH_2_PO_4_ supply treatments was conducted, which resulted in an average of 54.1 and 42.5 million clean reads in the libraries of stylo roots under LP and HP treatments, respectively (Additional file 2). From all samples, *de novo* assembly of the reads generated 326,640 transcripts and 173,014 unigenes (Additional file 2). The mean lengths of transcripts and unigenes were 787 and 1,243 bp, respectively (Additional file 2). A total of 8,348 genes with |log_2_(fold change)|≥1 and *q*<0.05 were considered as differentially expressed genes (DEGs) in stylo roots from the two P treatments (Additional file 3). Among them, 3,707 and 4,641 genes were up-regulated and down-regulated by Pi starvation in stylo roots, respectively (Additional file 3). Subsequently, a hierarchical clustering of all DEGs was generated to show the overview of the transcriptomic profiles (Additional file 4). The DEGs were clustered into three main clusters under the two P treatments (Additional file 4). Cluster 1 included both the up-regulated and down-regulated DEGs, while the expression levels of DEGs in clusters 2 and 3 were decreased and increased by LP treatment, respectively, compared to the HP treatment (Additional file 4).

**Fig. 6:**
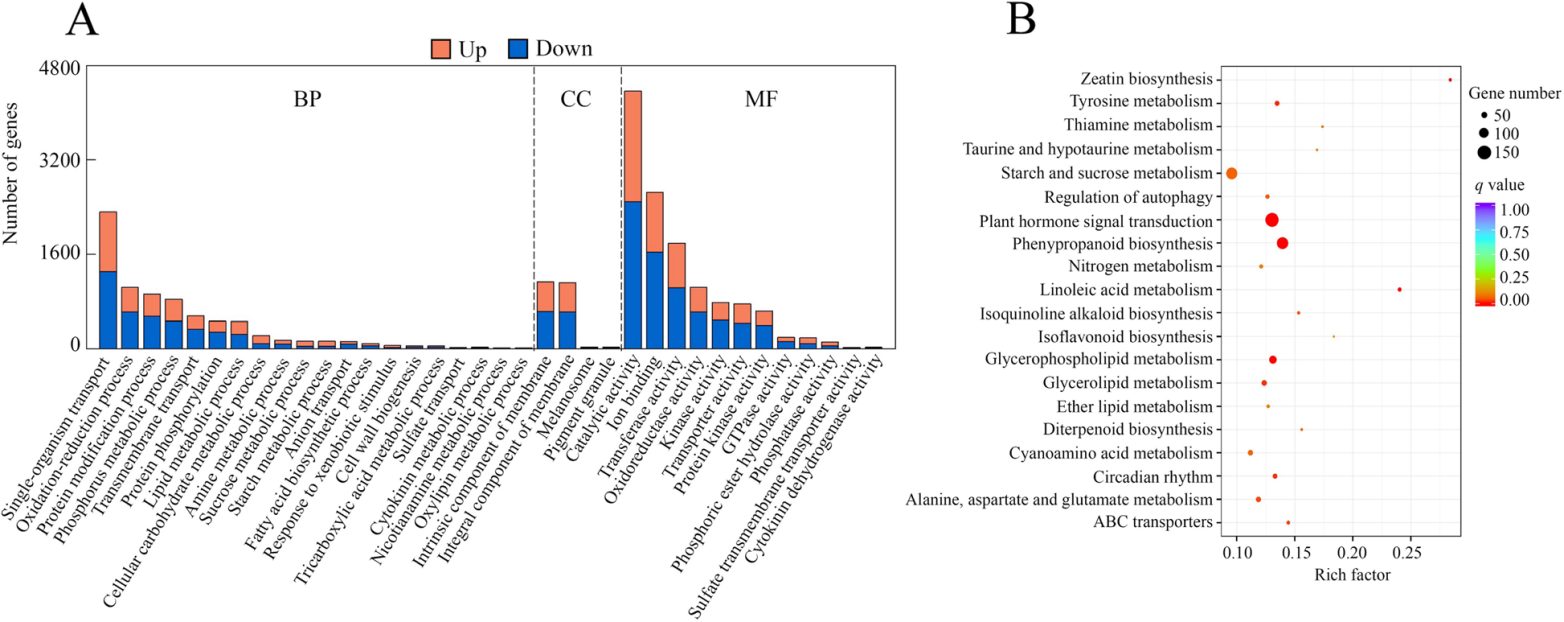
GO and KEGG analysis of DEGs. **a** GO terms associated with biological process (BP), cellular component (CC) and molecular function (MF). **b** KEGG enrichment analysis of DEGs regulated by P deficiency in stylo roots. KEGG analysis was analyzed according to KEGG database (https://www.kegg.jp/)

Gene Ontology (GO) analysis showed that the identified DEGs can be classified into biological processes (BP), cellular components (CC) and molecular function (MF), which included 20, 4 and 12 GO terms, respectively (Fig. 6a). Among the BP group, single-organism metabolic process, oxidation-reduction process, protein modification process, phosphorus metabolic process, transmembrane transport, protein phosphorylation and lipid metabolic process were the main terms (Fig. 6a). The dominant terms in CC contained intrinsic component of membrane, integral component of membrane, melanosome and pigment granule (Fig. 6a). Among the MF group, catalytic activity, ion binding, transferase activity, oxidoreductase activity, kinase activity, transporter activity and protein kinase activity were the major terms (Fig. 6a). Furthermore, the identified DEGs were analyzed via the Kyoto Encyclopedia of Genes and Genomes (KEGG) functional enrichment. There were 25 significantly enriched KEGG pathways. Among them, plant hormone signal transduction, phenylpropanoid biosynthesis, starch and sucrose metabolism, glycerophospholipid metabolism, glycerolipid metabolism, alanine, aspartate and glutamate metabolism, cyanoamino acid metabolism, tyrosine metabolism, circadian rhythm-plant and inositol phosphate metabolism were the most dominant KEGG pathways (Fig. 6b).

### Transporters in response to low P availability

**Fig. 7:**
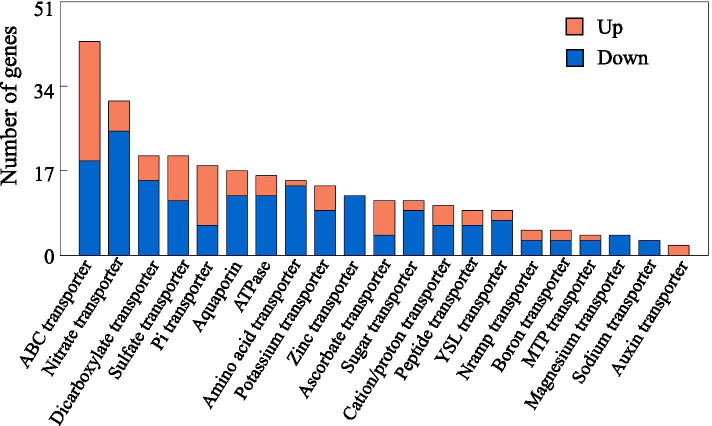
DEGs related to transporters in stylo roots. X- and Y-axis indicate different kinds of transporters and their corresponding number, respectively

**Fig. 8:**
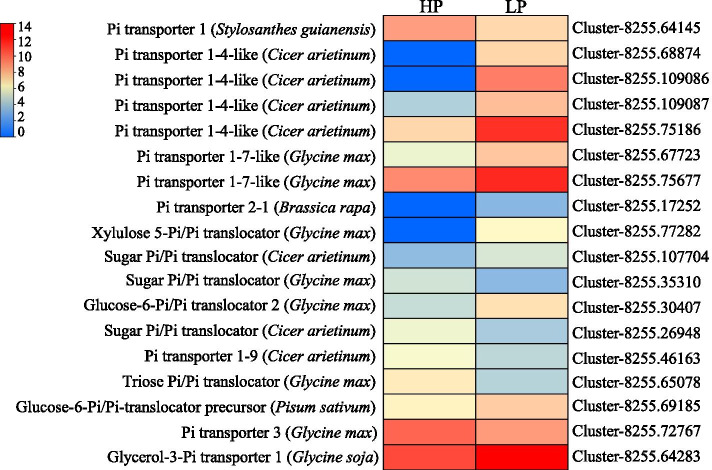
Heatmap visualization of the expression changes of the DEGs related to Pi transporters. The transcripts of DEGs were normalized as log_2_(FPKM+1). Gene IDs are showed by the legend on the right. Expression levels ranging from red to blue indicate high to low expression for genes, respectively. LP and HP represent 0 and 250 μM KH_2_PO_4_ supply treatments, respectively

A total of 279 DEGs encoding different kinds of transporters were enriched based on GO analysis (Additional file 5). The most significant enriched transporter genes were classified into ABC transporter, nitrate transporter, dicarboxylate transporter, sulfate transporter and Pi transporter (Fig. 7 and Additional file 5). For example, 43 genes were identified as ABC transporter, including 24 up-regulated and 19 down-regulated genes (Fig. 7 and Additional file 5). The transcripts of *ABC transporter D family member 1*, *ABC transporter B family member 26*, *ABC transporter B family member 19* and *ABC transporter C family member 3* were induced by low-P stress, while *ABC transporter ATP-binding protein* was completely suppressed (Additional file 5). Interestingly, 25 out of 31 genes encoding nitrate transporter were down-regulated by P deficiency, but six genes were enhanced, such as *NRT1/PTR FAMILY 8.3*, *nitrate transporter 1.4-like*, *putative nitrate excretion transporter 2*, *probable peptide/nitrate transporter At5g62680-like*, and two *probable nitrate transporter At1g68570* (Additional file 5). Among the 18 DEGs belonging to the putative Pi transporter, 12 and 6 genes were up-regulated and down-regulated by Pi starvation, respectively (Fig. 8). For example, transcripts of two *inorganic phosphate transporter 1-4-like*, one *xylulose 5-phosphate/phosphate translocator* and one *inorganic phosphate transporter 2-1* were significantly induced by low P treatment (Additional file 5). In addition, a set of 20 genes enriched in dicarboxylate transporter were found to be mainly encoding for multidrug and toxin extrusion (MATE) and aluminum-activated malate transporter (ALMT) (Additional file 5). Among them, five *MATE* homologues, including one *MATE efflux family protein 5-like*, one *MATE efflux family protein 8-like*, two *MATE efflux family protein 5* and one *MATE efflux family protein DTX1-like*, were up-regulated by P deficiency, and three genes encoding ALMT family protein were down-regulated, such as *aluminum-activated malate transporter 10-like*, *aluminum-activated malate transporter 9-like* and *aluminum-activated malate transporter 2* (Additional file 5).

### DEGs related to phosphatases and root growth responding to P deficiency

**Fig. 9:**
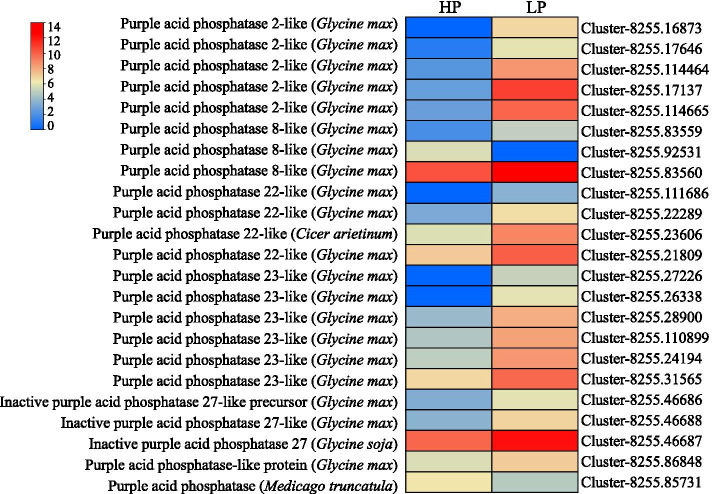
Heatmap analysis of the DEGs associated with purple acid phosphatases. The transcripts of DEGs were normalized as log_2_(FPKM+1). Gene IDs are showed by the legend on the right. Expression levels ranging from red to blue indicate high to low expression for genes, respectively. LP and HP represent 0 and 250 μM KH_2_PO_4_ supply treatments, respectively

A total of 116 DEGs were predicted to encode phosphatases according to GO analysis, including 68 up-regulated genes and 48 down-regulated genes (Additional file 6). Among them, a group of 23 genes were related to putative purple acid phosphatases (PAP) (Fig. 9 and Additional file 6). As shown in Fig. 9, 21 *PAP* homologues were up-regulated by P deficiency, and only two *PAPs* were down-regulated in stylo roots. Among them, DEGs, such as *purple acid phosphatase 22-like*, *purple acid phosphatase 2-like* and *purple acid phosphatase 23-like* homologues were induced by Pi starvation, while *purple acid phosphatase 8-like* was suppressed (Fig. 9).

In addition, twelve genes related to root growth were also found to be regulated by P deficiency, including five up-regulated and seven down-regulated genes. Among them, one *expansin-A13* and two *expansin-B1-like* genes were up-regulated and down-regulated by P deficiency, respectively (Additional file 7).

### Potential transcription factors and regulators involved in Pi signaling

**Fig. 10:**
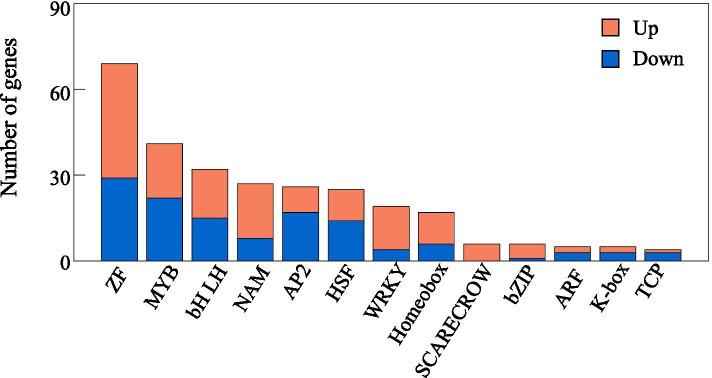
DEGs related to transcription factors. X- and Y-axis indicate different kinds of TF and their corresponding number, respectively

A total of 282 DEGs were annotated as transcription factors (TFs) (Fig. 10 and Additional file 8). Among them, genes encoding zinc finger (ZF) TFs were the largest group, containing 29 up-regulated and 40 down-regulated genes. The other TFs included genes encoding MYB (41), bHLH (32), NAM (27), AP2 (26), HSF (25), WRKY (19), Homeobox (17), SCARECROW (6), bZIP (6), ARF (5), K-box (5) and TCP (4) families (Fig. 10). Among the ZF TFs, six genes were induced and four genes were absolutely suppressed by P deficiency (Additional file 8). Furthermore, four ZF genes including *zinc finger protein NUTCRACKER-like, zinc finger BED domain-containing protein RICESLEEPER 2-like, zinc finger CCCH domain protein* and *DHHC-type zinc finger protein* were up-regulated by more than 6-fold under P deficiency, whereas *zinc finger protein JACKDAW-like* was suppressed by more than 6-fold under P deficiency (Additional file 8). There were 41 genes belonging to MYB TFs, including 22 up-regulated and 19 down-regulated genes. Among these MYB TFs, two *PHR1-like* homologues were down-regulated by P deficiency (Additional file 8). Interestingly, a total of 63 TFs were predicted to be involved in plant hormone signal transduction, including genes associated with ethylene, auxin, abscisic acid and brassinosteroid signaling pathway (Additional file 9). In addition, genes encoding protein containing SPX domain were also found to be regulated by P deficiency. Among them, 9 out of 13 *SPX* homologues were up-regulated by more than 8-fold under P deficiency, and the remaining four *SPXs* were down-regulated in stylo roots (Additional file 10).

**Fig. 11:**
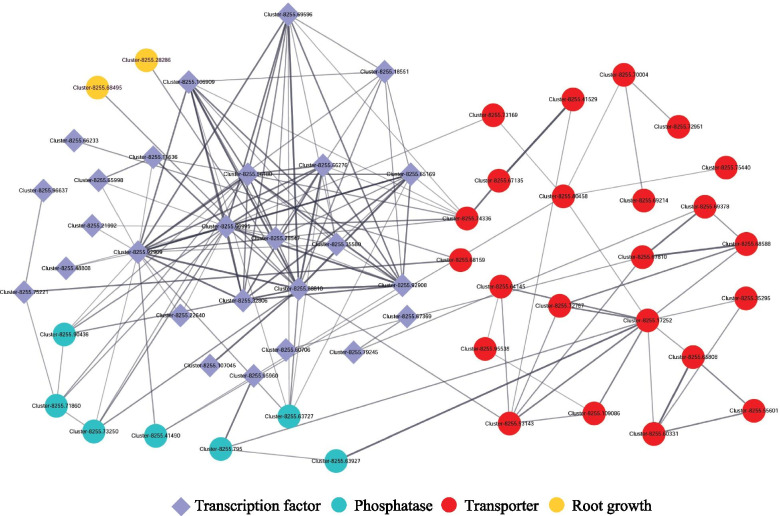
Interaction networks of genes associated with transporters, phosphatase, root growth and transcription factor. Gene information is available in Additional file 3: Table S3. Cytoscape (version 3.8.0) was used to analyze the interaction networks

Subsequently, the interaction networks were further constructed to explore the candidate TFs potentially involved in the regulation of genes related to transporters, phosphatase and root growth in stylo during P deficiency. Results showed that the interaction networks contained 147 edges with 58 nodes (Fig. 11). Among them, one gene encoding ATPase subunit 1 (Cluster-8255.74336) was regulated by seven TFs, including five homologues of *heat shock protein* (Cluster-8255.65169, Cluster-8255.66995, Cluster-8255.56180, Cluster-8255.106909 and Cluster-8255.92909) and two *probable mediator of RNA polymerase II transcription subunit 37c-like* (Cluster-8255.69596 and Cluster-8255.66276) (Fig. 11). One *high-affinity nitrate transporter 3.1-like* (Cluster-8255.67810) was found to be regulated by one TF belonging to WRKY family (Cluster-8255.60706). Interestingly, two root growth related genes, *protein ROOT HAIR DEFECTIVE 3 homolog 1-like* (Cluster-8255.68495) and *protein ROOT HAIR DEFECTIVE 3 homolog 2-like* (Cluster-8255.28286), were both regulated by *chaperone protein dnaJ* (Cluster-8255.88810). Furthermore, *heat shock 70 kDa protein 16-like isoform X1* (Cluster-8255.92909) and *heat shock protein 83 isoform X2* (Cluster-8255.66995), belonging to HSF family, were found to target to 21 and 20 genes in the interaction networks, respectively (Fig. 11). These results suggest the complex nature of regulatory networks of stylo in response to P deficiency.

### Analysis of RNA-seq data using quantitative real-time PCR (qRT-PCR)

A total of 13 DEGs from the RNA-seq data, including 9 up-regulated and 4 down-regulated genes, were selected for qRT-PCR analysis in RY2 roots at two P levels. Results showed that the trends of transcripts of the tested genes based on qRT-PCR matched well with its corresponding expression presented in the RNA-seq data, as reflected by a significant correlation (R^2^=0.811, *P*<0.01) (Additional file 11).

## Discussion

P deficiency influences numerous biological processes in crop plants, such as rice, soybean and wheat, markedly reducing crop yield [40–42]. In this study, differences in low-P tolerance were observed between the two stylo cultivars, and RY2 is identified as a low-P tolerant cultivar. Stylo RY2 exhibited lower rates of decrease in growth parameters than RY5 under low-P stress, especially for shoot and root dry weight (Figs. 1 and 2). Furthermore, P acquisition efficiency of RY2 was higher than that of RY5 under the same P treatment (Fig. 3). It has been well demonstrated that changes in root system architecture increase P acquisition by plants from the soil, and these root traits include larger values of root biomass, root/shoot ratio and root surface area in the topsoil [11–13]. For example, increases in root/shoot biomass ratio are observed in maize and wheat, while higher specific root length is found in rapeseed under low P supply, which are considered adaptive strategies beneficial for improving P acquisition [13]. Similarly, root growth parameters including total root length, lateral root length and root surface area, were higher in RY2 than those in RY5 under low-P stress (Fig. 4). Thus, the stylo cultivar RY2 is more tolerant to P deficiency probably through regulation of its root growth.

To elucidate the molecular mechanisms underlying the adaptation of stylo to P deficiency, overall transcriptome changes in roots of cultivar RY2 in response to P deficiency were investigated using RNA-seq analysis. Many of the identified DEGs were found to encode putative transporters, phosphatases and transcription factors that are associated with root growth, Pi acquisition and Pi homeostasis as well as Pi signaling. Among them, a group of DEGs were categorized as transporters, such as nitrate transporter, Pi transporter and dicarboxylate transporter (Additional file 5). Many genes encoding nitrate transporters were regulated by P deficiency, including six low P-enhanced *nitrate transporter* genes (Additional file 5). Regulation of genes encoding nitrate transporters by P deficiency has also been observed in Arabidopsis [43], rice [44] and soybean [45]. In addition to function in enhancing nitrogen (N) use efficiency, overexpression of the *high-affinity nitrate transporter*, *OsNRT2.3b*, can increase P uptake and accumulation in rice through regulation of root growth and expression of PSI genes (e.g., *OsPT2*, *OsPT8* and *OsPHR2*) [46]. Interestingly, four putative *PT* homologues encoding inorganic phosphate transporter 1-4-like, two *PT* genes encoding inorganic phosphate transporter 1-7-like and one member belonging to inorganic phosphate transporter 2-1 were up-regulated by P deficiency in this study (Fig. 8). Numerous *PT* homologues are found to be enhanced by P deficiency in soybean [23], rice [47] and Medicago (*Medicago truncatula*) [48]. For example, in Arabidopsis, the transcripts of two *Pi transporters* (*Pht1;1* and *Pht1;4*) are significantly increased in low P-treated roots, which are involved in Pi uptake [49, 50]. In rice, *OsPT2* is up-regulated in both shoots and roots by low-P stress, and is found to be involved in Pi acquisition and the long-distance translocation of Pi from roots to shoots [51]. Therefore, these results suggest that the low P up-regulated nitrate transporter and Pi transporter can regulate Pi transport and Pi homeostasis in stylo as part of the underlying molecular mechanism of adaptation to P deficiency, which deserves further investigation.

As a large amount of soil P is poorly available for plants, one of the important strategies for increasing P mobilization and uptake is to stimulate the release of Pi from sparingly soluble P through secreted organic acids from root to rhizosphere [52]. Transporters responsible for organic acid transport, such as ALMT and MATE, have been well demonstrated to be participating in malate and citrate transport, respectively [52, 53]. Divergent expression patterns of *ALMTs* or *MATEs* are found in plants response to abiotic stresses, such as salt and proton stresses, P deficiency and Al toxicity [53–55]. In this study, the up-regulation of *MATE* homologues probably results in increasing citrate exudation from stylo roots in response to P deficiency, which can be supported by the observation of citrate secreted from RY2 roots under P deficient condition [56]. As genes encoding putative malate transporter proteins were suppressed by P deficiency (Additional file 5), the *MATE* genes mediated citrate exudation are possibly involved in P mobilization in stylo, and their functions merit further characterization.

Enhancing transcription and activity of phosphatases is generally considered to be helpful for plants increasing Pi availability by hydrolyzed organic P to release Pi [37, 38]. A set of genes encoding phosphatases were found to be differentially expressed under two P levels in this study (Additional file 6). Interestingly, 21 out of 23 *PAP* homologues, such as *purple acid phosphatase 2-like*, *purple acid phosphatase 8-like*, *purple acid phosphatase 22-like*, *purple acid phosphatase 23-like* and *purple acid phosphatase 27-like*, were up-regulated by Pi starvation in stylo roots; this was consistent with the increasing APase activity in stylo roots under P deficient treatment (Figs. 5 and 9). Similarly, 10 out of 26 *PAP* members in rice, 11 out of 29 *PAPs* in Arabidopsis, 23 out of 35 *PAPs* in soybean and 12 out of 25 *PAPs* in chickpea are known to be up-regulated by P deficiency, potentially participating in organic P utilization [15, 57–59]. Therefore, it is reasonable to assume that the Pi starvation-enhanced *PAP* homologues are possibly involved in organic P utilization by stylo to cope with P deficiency.

Optimizing root growth parameters, such as total root length, lateral root branching and root hair density, is particularly important for plants to increase Pi acquisition. A variety of genes have been reported to be responsible for root growth, including *expansin* genes. For example, *GmEXPB2*, an *expansin* gene in soybean, is mainly expressed in roots and is induced by P deficiency; functional analysis demonstrates that *GmEXPB2* is involved in root cell division and root hair elongation, thereby contributing to plant growth and P uptake [30]. In this study, three *expansin* homologues were found to be differentially regulated by P deficiency (Additional file 7). A similar result has been recently reported [60] in which seven *expansin* genes are found to be regulated by P deficiency in stylo, including six up-regulated and one down-regulated genes and these genes are suggested to be involved in stylo root growth under Pi deprivation. Therefore, these results suggest that some of the stylo *expansin* genes may be participating in modifying root growth adaptation to P deficiency, but their exact involvement needs to be further studied.

The adaptive strategies employed by plants to maintain cellular Pi homeostasis are generally regulated by signaling network involving transcription factors and other potential regulators [18]. Several TFs have been demonstrated as key participants in Pi signaling pathways, such as members belonging to MYB, WRKY, bHLH and ARF families, which were also identified in this study (Fig. 10 and Additional file 8). For example, PHR1 belonging to the MYB family, is the most well-characterized TF and also a central integrator in transcriptional regulation of Pi starvation responses (PSR). A variety of PHR1 homologues with similar functions in regulating Pi homeostasis have been identified in many plants, such as AtPHR1, PHR1-like1 (PHL1) and PHL2 in Arabidopsis, OsPHR1/2/3/4 in rice, GmPHR25 in soybean and PvPHR1 in common bean, and most of these *PHR1* homologues are up-regulated by P deficiency [8, 61–64]. In this study, a total of 41 DEGs were identified as TFs belonging to MYB family. Among them, two *PHR1-like* homologues were found to be down-regulated by P deficiency (Additional file 8), suggesting that there are other regulatory mechanisms mediated by *PHR1* in the adaptation of stylo to P deficiency in soil, which are probably different from those well-known PHR1 members identified in other plants. In addition, a set of TFs belonging to WRKY family were found to be regulated by P deficiency in stylo roots (Additional file 8). WRKY is an important TF involved in Pi signaling pathways. It has been reported that WRKY6 and WRKY75 in Arabidopsis are involved in regulating PSI genes, such as genes encoding phosphatases and high-affinity Pi transporters, controlling Pi homeostasis and root growth in response to P deficiency [65, 66]. Interestingly, in this study, one *high-affinity nitrate transporter 3.1-like* (Cluster-8255.67810) was found to be regulated by one WRKY member (Cluster-8255.60706) (Fig. 11), suggesting a possible dual role for these genes in regulation of Pi as well as N homeostasis.

In this study, 13 DEGs related to the putative SPX domain-containing proteins involved in Pi signaling pathways were found to be regulated by P deficiency (Additional file 10). Similar results are also found in soybean where *GmSPXs* exhibited differential regulation by P deficiency in soybean root [67]. SPX domain-containing proteins have been well characterized to be the important components in Pi signaling pathway in Arabidopsis, rice and soybean [67–69]. For example, *OsSPX4* acts as a negative regulator of *OsPHR2*. *OsSPX4* could interact with *OsPHR2* through inhibition of the binding of *OsPHR2* to its target genes under Pi sufficient condition, whereas OsSPX4 can be degraded through the 26S proteasome pathway under Pi deficiency [70]. Therefore, the identified various TFs and regulators from this study suggest a complex response mechanism of stylo to P deficiency, which in turn contributes to maintain cellular Pi homeostasis.

## Conclusion

This study revealed that P deficiency resulted in reduction of stylo growth, and stylo cultivar RY2 exhibited higher low-P tolerance than RY5. Numerous DEGs were identified through transcriptomic analysis of RY2 roots under P deficiency. Furthermore, DEGs encoding putative transporters, phosphatases and transcription factors suggested the underlying mechanisms of adaptation of stylo to P deficiency. The low-P tolerance of RY2 is probably manifested through regulation of root growth, Pi acquisition and cellular Pi homeostasis as well as Pi signaling pathway. The identified genes can be potentially used to design a breeding strategy for developing P-efficient stylo cultivars to grow on acid soils in the tropics.

## Methods

### Plant growth and treatments

In this study, two stylo (*Stylosanthes guianensis*) cultivars, ‘RY2’ and ‘RY5’, were used, which were widely grown in South China [33]. The stylo seeds were provided by the Institute of Tropical Crop Genetic Resources (TCGRI), Chinese Academy of Tropical Agricultural Sciences (CATAS), Hainan, China. Experiments were performed in a greenhouse at temperatures of 25 °C to 32 °C under natural sunlight with a photoperiod of about 13 h at the TCGRI, CATAS, Hainan, China (19°30′N, 109°30′E). Seeds were germinated for 3 d, and stylo seedlings were then transferred to a modified Hoagland nutrient solution containing 250 μM KH_2_PO_4_ for 14 d as previously described [71]. After that, seedlings were separately transplanted into nutrient solution supplied with 0, 100 and 250 μM KH_2_PO_4_, which were regarded as low (Pi deprivation), moderate and high P supply treatments, respectively. The nutrient solution was adjusted to a pH value of 5.8 and refreshed weekly. After 21 d of P treatments, shoots and roots were separately harvested for further analysis. An individual hydroponic box containing three seedlings of each stylo cultivar was set as one biological replicate. Each treatment included three biological replicates.

### Determination of root morphology and P concentration

Plant fresh roots were scanned using an Epson 12000XL scanner (Epson, Japan) with a resolution of 300 dpi, and the obtained image was saved as JEPG format. Total root length, root surface area and root volume were analyzed with WinRhizo Pro software (Regent Instruments Inc., Quebec, Canada). After that, shoot and root samples were oven dried at 75 °C for 7 d, and the dry mass of shoots and roots was further determined. For P concentration analysis, approximately 0.07 g dry samples were burned to ash at 600 °C in a muffle furnace. The sample of ash was absolutely dissolved in 100 mM HCl, and the supernatant was then used for P concentration analysis as previously described [72].

### Analysis of APase activity

APase activities in stylo leaf and root were analyzed as previously described [38] with some modification. Approximately 0.15 g of leaf and root samples were ground in 1.5 mL of 45 mM Na-acetate buffer (pH 5.0) at 4 °C. After centrifugation at 12,000 rpm for 15 min at 4 °C, the supernatants were mixed with 2 mL of 45 mM Na-acetate buffer (pH 5.0) containing 1 mM ρ-nitrophenyl phosphate (Sigma, Saint Louis, MO, USA). After incubation at 37 °C for 15 min, the reaction was terminated by the addition of 1 mL of 1 M NaOH. APase activity was spectrophotometrically detected at 405 nm and expressed as micromoles of ρ-nitrophenyl phosphate hydrolyzed per mg protein per min. Protein concentration in the extracts was analyzed using the Coomassie Brilliant Blue staining method [73].

### RNA extraction and sequencing

Total RNA from roots of RY2 at 0 (LP) and 250 (HP) μM KH_2_PO_4_ treatments was isolated using Trizol reagent (Invitrogen, Carlsbad, CA, USA) following the manufacturer's instructions. RNA purity and integrity were assessed by Nanodrop 2000c Spectrophotometer (Thermo Fisher Scientific, Waltham, MA, USA) and Agilent 2100 (Agilent Technologies, Palo Alto, CA, USA), respectively. RNA sequencing analysis was conducted by Novogene Bioinformatics Technology Co., Ltd. (Beijing, China). RNA-seq libraries were constructed using the NEBNext® Ultra^TM^ RNA Library Prep kit (NEB, Beverly, MA, USA), and the cDNA libraries were sequenced using an Illumina Hiseq^TM^ platform (Illumina, San Diego, CA, USA). The 150-bp paired-end reads (PE150) were generated.

### Transcriptomic analysis

RNA-seq raw data were obtained using the Casava v.1.8 program. The raw reads in FASTQ format were processed, and then the high-quality clean reads were obtained after removing adaptor, ploy-N and low-quality sequences. The final clean reads were assembled using Trinity software (version 2). For annotation, all assembled unigenes were searched against a number of public databases, including the National Center for Biotechnology Information (NCBI) non-redundant protein sequences (Nr), the non-redundant nucleotide sequences (Nt), the Protein Family (Pfam), the Clusters of Orthologous Groups of protein database (COG), the Swiss-Prot protein database, the GO and the KEGG databases.

The expression level of each gene was analyzed and represented by the expected number of fragments per kilobase of transcript sequence per millions base pairs (FPKM) using RSEM software with default settings [74]. Differentially expressed genes between two P treatments were identified using DESeq2 [75]. Genes with *q*-value <0.05 and |log_2_(fold change)| ≥1 were assigned as differentially expressed. GO and KEGG enrichment analyses of DEGs were performed as previously described [71, 76]. The interaction networks were analyzed by Cytoscape (version 3.8.0). The raw data were deposited in the Gene Expression Omnibus under GEO series number GSE171448.

### Validation of DEGs by qRT-PCR analysis

A total of 13 DEGs were selected to assess the accuracy of RNA-seq data using qRT-PCR method. qRT-PCR analysis was performed according to SYBR Green Master Mix kit (Vazyme, China), and was monitored on a QuantStudio™ 6 Flex Real-Time System (Thermo Fisher Scientific, Waltham, MA, USA). Specific primers of the tested genes are listed in Additional file 12. The relative expression of candidate gene was calculated relative to the expression of reference gene *SgEF-1a* as previously described [37]. Three biological replicates were included in the qRT-PCR analysis.

### Statistical analysis

Data analysis was performed for the mean and standard error calculation using Microsoft Excel 2003 (Microsoft Company, USA). One-way ANOVA and Student’s *t*-test analyses were performed with the SPSS program (SPSS Institute, USA, v. 13.0).

## Supplementary Information

**Additional file 1: Table S1.** The decrease rate of growth parameters of stylo under 0 (LP) and 100 μM KH_2_PO_4_ relative to that in 250 (HP) μM KH_2_PO_4_ treatment.

**Additional file 2: Table S2.** Summary of stylo roots transcriptomes in 0 (LP) and 250 (HP) μM KH_2_PO_4_ supply treatments.

**Additional file 3: Table S3.** DEGs identified in stylo roots under 0 (LP) and 250 (HP) μM KH2PO4 supply treatments.

**Additional file 4: Figure S1.** Clustering analysis of the DEGs under two P conditions. The transcripts of DEGs were normalized as log_2_(FPKM). Expression levels ranged from red to blue indicate high to low expression for genes, respectively. LP and HP represent 0 and 250 μM KH_2_PO_4_ supply treatments, respectively.

**Additional file 5: Table S4.** DEGs related to transporters.

**Additional file 6: Table S5.** DEGs encoding putative phosphatases.

**Additional file 7: Table S6.** DEGs related to root growth.

**Additional file 8: Table S7.** Potential transcription factors involved in Pi signaling.

**Additional file 9: Table S8**. DEGs related to plant hormone signal transduction.

**Additional file 10: Figure S2.** Heatmap analysis of the DEGs belonging to SPX containing proteins. The transcripts of DEGs were normalized as log2(FPKM+1). Gene IDs were showed by the legend on the right. Expression levels ranged from red to blue indicate high to low expression for genes, respectively. LP and HP represent 0 and 250 μM KH_2_PO_4_ supply treatments, respectively.

**Additional file 11: Figure S3.** Correlation analysis of gene expression between transcriptome data and qRT-PCR results. Nine up-regulated and four down-regulated DEGs were selected for qRT-PCR analysis. Transcriptome data were plotted against data from qRT-PCR. Data are presented on a log_2_ scale.

**Additional file 12: Table S9.** Primers used for qRT-PCR analysis of the selected genes.

## Data Availability

The datasets are included in this article and its Additional files are available from the corresponding author on reasonable request. The datasets for this study can be found in the NCBI Gene Expression Omnibus under GEO series number of GSE171448.

## Abbreviations

ACP: Acid phosphatase
ALMT: Aluminum-activated malate transporter
ATP: Adenosine triphosphate
BP: Biological processes
CC: Cellular components
COG: Clusters of orthologous groups of protein database
DEGs: Differentially expressed genes
dNTP: Deoxynucleoside triphosphate
FPKM: Expected number of fragments per kilobase of transcript sequence per millions base pairs
GO: Gene ontology
KEGG: Kyoto encyclopedia of genes and genomes
MATE: Multidrug and toxin extrusion
MF: Molecular function
NCBI: National center for biotechnology information
Nr: Non-redundant protein sequences
Nt: Non-redundant nucleotide sequences
PAP: Purple acid phosphatase
Pfam: Protein family
PHR1: PHOSPHATE STARVATION RESPONSE 1
PPCK: Phosphoenolpyruvate carboxylase kinase
PSI: Pi starvation induced
PSR: Pi starvation responses
PT: Pi transporter
qRT-PCR: Quantitative real-time polymerase chain reaction
RNA-seq: RNA sequencing
RNS: Ribonuclease
SPX: SYG1/PHO81/XPR1
TFs: Transcription factors

## References

[1] Raghothama KG (1999). Phosphate acquisition. Annu Rev Plant Physiol Plant Mol Biol.

[2] Mora-Macías J, Ojeda-Rivera JO, Gutiérrez-Alanís D, Yong-Villalobos L, Oropeza-Aburto A, Raya-González J (2017). Malate-dependent Fe accumulation is a critical checkpoint in the root developmental response to low phosphate. Proc Natl Acad Sci U S A.

[3] Ao JH, Chen ZJ, Wu M, Lu X, Huang Z, Liao H (2014). Phosphorus fractions of red soils in Guangdong province of South China and their bioavailability for five crop species. Soil Sci.

[4] Kochian LV, Piñeros MA, Liu J, Magalhaes JV (2015). Plant adaptation to acid soils: The molecular basis for crop aluminum resistance. Annu Rev Plant Biol.

[5] Conley DJ, Paerl HW, Howarth RW, Boesch DF, Seitzinger SP, Havens KE (2009). Controlling eutrophication: nitrogen and phosphorus. Science..

[6] Cordell D, Drangert J, White S (2009). The story of phosphorus: global food security and food for thought. Glob Environ Chang.

[7] Wu P, Shou H, Xu G, Lian X (2013). Improvement of phosphorus efficiency in rice on the basis of understanding phosphate signaling and homeostasis. Curr Opin Plant Biol.

[8] Ham BK, Chen J, Yan Y, Lucas WJ (2018). Insights into plant phosphate sensing and signaling. Curr Opin Biotechnol.

[9] Lynch JP (2011). Root phenes for enhanced soil exploration and phosphorus acquisition: tools for future crops. Plant Physiol.

[10] Rao IM, Miles JW, Beebe SE, Horst WJ (2016). Root adaptations to soils with low fertility and aluminium toxicity. Ann Bot.

[11] Bayuelo-Jiménez JS, Gallardo-Valdéz M, Pérez-Decelis VA, Magdaleno-Armas L, Ochoa I, Lynch JP (2011). Genotypic variation for root traits of maize (*Zea mays L.*) from the Purhepecha Plateau under contrasting phosphorus availability. Field Crop Res.

[12] Liu HT, White PJ, Li CJ (2016). Biomass partitioning and rhizosphere responses of maize and faba bean to phosphorus deficiency. Crop Pasture Sci.

[13] Lyu Y, Tang H, Li H, Zhang F, Rengel Z, Whalley WR (2016). Major crop species show differential balance between root morphological and physiological responses to variable phosphorus supply. Front Plant Sci.

[14] Mehra P, Pandey BK, Giri J (2017). Improvement in phosphate acquisition and utilization by a secretory purple acid phosphatase (OsPAP21b) in rice. Plant Biotechnol J.

[15] Bhadouria J, Singh AP, Mehra P, Verma L, Srivastawa R, Parida SK (2017). Identification of purple acid phosphatases in chickpea and potential roles of *CaPAP7* in seed phytate accumulation. Sci Rep.

[16] Zhu S, Chen M, Liang C, Xue Y, Lin S, Tian J (2020). Characterization of purple acid phosphatase family and functional analysis of *GmPAP7a/7b* involved in extracellular ATP utilization in soybean. Front Plant Sci.

[17] Liang C, Wang J, Zhao J, Tian J, Liao H (2014). Control of phosphate homeostasis through gene regulation in crops. Curr Opin Plant Biol.

[18] Chiou TJ, Lin SI (2011). Signaling network in sensing phosphate availability in plants. Annu Rev Plant Biol.

[19] Mitsukawa N, Okumura S, Shirano Y, Sato S, Kato T, Harashima S (1997). Overexpression of an *Arabidopsis thaliana* high-affinity phosphate transporter gene in tobacco cultured cells enhances cell growth under phosphate-limited conditions. Proc Natl Acad Sci U S A.

[20] Mudge SR, Rae AL, Diatloff E, Smith FW (2002). Expression analysis suggests novel roles for members of the Pht1 family of phosphate transporters in Arabidopsis. Plant J.

[21] Sun S, Gu M, Cao Y, Huang X, Zhang X, Ai P (2012). A constitutive expressed phosphate transporter, OsPht1;1, modulates phosphate uptake and translocation in phosphate-replete rice. Plant Physiol.

[22] Wang X, Wang Y, Piñeros MA, Wang Z, Wang W, Li C (2014). Phosphate transporters OsPHT1;9 and OsPHT1;10 are involved in phosphate uptake in rice. Plant Cell Environ.

[23] Qin L, Zhao J, Tian J, Chen L, Sun Z, Guo Y (2012). The high-affinity phosphate transporter GmPT5 regulates phosphate transport to nodules and nodulation in soybean. Plant Physiol.

[24] Chen L, Qin L, Zhou L, Li X, Chen Z, Sun L (2019). A nodule-localized phosphate transporter GmPT7 plays an important role in enhancing symbiotic N2 fixation and yield in soybean. New Phytol.

[25] Wang L, Lu S, Zhang Y, Li Z, Du X, Liu D (2014). Comparative genetic analysis of Arabidopsis purple acid phosphatases AtPAP10, AtPAP12, and AtPAP26 provides new insights into their roles in plant adaptation to phosphate deprivation. J Integr Plant Biol.

[26] Li RJ, Lu WJ, Guo CJ, Li XJ, Gu JT, Xiao K (2012). Molecular characterization and functional analysis of *OsPHY1*, a purple acid phosphatase (PAP)-type phytase gene in rice (*Oryza sativa* L.). J Integr Plant Biol.

[27] Liang C, Sun L, Yao Z, Liao H, Tian J (2012). Comparative analysis of *PvPAP* gene family and their functions in response to phosphorus deficiency in common bean. PLoS One.

[28] Kong Y, Li X, Wang B, Li W, Du H, Zhang C (2018). The soybean purple acid phosphatase GmPAP14 predominantly enhances external phytate utilization in plants. Front Plant Sci.

[29] Li C, Zhou J, Wang X, Liao H (2019). A purple acid phosphatase, *GmPAP33*, participates in arbuscule degeneration during arbuscular mycorrhizal symbiosis in soybean. Plant Cell Environ.

[30] Guo W, Zhao J, Li X, Qin L, Yan X, Liao H (2011). A soybean β-expansin gene *GmEXPB2* intrinsically involved in root system architecture responses to abiotic stresses. Plant J.

[31] Yu Z, Kang B, He X, Lv S, Bai Y, Ding W (2011). Root hair-specific expansins modulate root hair elongation in rice. Plant J.

[32] Chandra A (2009). Diversity among *Stylosanthes* species: habitat, edaphic and agro-climatic affinities leading to cultivar development. J Environ Biol.

[33] Liu GD, Bai CJ, Wang DJ, He HX, Chakraborty S (2004). *Stylosanthes* cultivars in China: their development and performance. High-yielding anthracnose-resistant *Stylosanthes* for agricultural systems.

[34] Guo PF, Liu PD, Lei J, Chen CH, Qiu H, Liu GD (2019). Improvement of plant regeneration and *Agrobacterium*-mediated genetic transformation of *Stylosanthes guianensis*. Trop Grassl Forrajes.

[35] Sun L, Liang C, Chen Z, Liu P, Tian J, Liu G (2014). Superior aluminium (Al) tolerance of *Stylosanthes* is achieved mainly by malate synthesis through an Al-enhanced malic enzyme, *SgME1*. New Phytol.

[36] Chen Z, Sun L, Liu P, Liu G, Tian J, Liao H (2015). Malate synthesis and secretion mediated by a manganese-enhanced malate dehydrogenase confers superior manganese tolerance in *Stylosanthes guianensis*. Plant Physiol.

[37] Liu P, Cai Z, Chen Z, Mo X, Ding X, Liang C (2018). A root-associated purple acid phosphatase, SgPAP23, mediates extracellular phytate-P utilization in *Stylosanthes guianensis*. Plant Cell Environ.

[38] Liu PD, Xue YB, Chen ZJ, Liu GD, Tian J (2016). Characterization of purple acid phosphatases involved in extracellular dNTP utilization in *Stylosanthes*. J Exp Bot.

[39] Du YM, Tian J, Liao H, Bai CJ, Yan XL, Liu GD (2009). Aluminium tolerance and high phosphorus efficiency helps *Stylosanthes* better adapt to low-P acid soils. Ann Bot.

[40] Gamuyao R, Chin JH, Pariasca-Tanaka J, Pesaresi P, Catausan S, Dalid C (2012). The protein kinase Pstol1 from traditional rice confers tolerance of phosphorus deficiency. Nature..

[41] Wu W, Lin Y, Liu P, Chen Q, Tian J, Liang C (2018). Association of extracellular dNTP utilization with a GmPAP1-like protein identified in cell wall proteomic analysis of soybean roots. J Exp Bot.

[42] Lin Y, Chen G, Hu H, Yang X, Zhang Z, Jiang X (2020). Phenotypic and genetic variation in phosphorus-deficiency-tolerance traits in Chinese wheat landraces. BMC Plant Biol.

[43] Lan P, Li WF, Schmidt W (2012). Complementary proteome and transcriptome profiling in phosphate-deficient Arabidopsis roots reveals multiple levels of gene regulation. Mol Cell Proteomics.

[44] Secco D, Jabnoune M, Walker H, Shou H, Wu P, Poirier Y (2013). Spatio-temporal transcript profiling of rice roots and shoots in response to phosphate starvation and recovery. Plant Cell.

[45] Zeng HQ, Wang GP, Zhang YQ, Hu XY, Pi EX, Zhu YY (2016). Genome-wide identification of phosphate-deficiency-responsive genes in soybean roots by high-throughput sequencin. Plant Soil.

[46] Feng H, Li B, Zhi Y, Chen J, Li R, Xia X (2017). Overexpression of the nitrate transporter, OsNRT2.3b, improves rice phosphorus uptake and translocation. Plant Cell Rep.

[47] Liu F, Chang XJ, Ye Y, Xie WB, Wu P, Lian XM (2011). Comprehensive sequence and whole-life-cycle expression profile analysis of the phosphate transporter gene family in rice. Mol Plant.

[48] Cao Y, Liu J, Li Y, Zhang J, Li S, An Y (2021). Functional analysis of the phosphate transporter gene *MtPT6* from *Medicago truncatula*. Front Plant Sci.

[49] Karthikeyan AS, Varadarajan DK, Mukatira UT, D'Urzo MP, Damsz B, Raghothama KG (2002). Regulated expression of Arabidopsis phosphate transporters. Plant Physiol.

[50] Shin H, Shin HS, Dewbre GR, Harrison MJ (2004). Phosphate transport in *Arabidopsis*: Pht1;1 and Pht1;4 play a major role in phosphate acquisition from both low- and high-phosphate environments. Plant J.

[51] Ai P, Sun S, Zhao J, Fan X, Xin W, Guo Q (2009). Two rice phosphate transporters, OsPht1;2 and OsPht1;6, have different functions and kinetic properties in uptake and translocation. Plant J.

[52] Peng W, Wu W, Peng J, Li J, Lin Y, Wang Y (2018). Characterization of the soybean GmALMT family genes and the function of GmALMT5 in response to phosphate starvation. J Integr Plant Biol.

[53] Magalhaes JV, Liu J, Guimarães CT, Lana UG, Alves VM, Wang YH (2007). A gene in the multidrug and toxic compound extrusion (*MATE*) family confers aluminum tolerance in sorghum. Nat Genet.

[54] Liang C, Piñeros MA, Tian J, Yao Z, Sun L, Liu J (2013). Low pH, aluminum, and phosphorus coordinately regulate malate exudation through *GmALMT1* to improve soybean adaptation to acid soils. Plant Physiol.

[55] Chen Q, Wang L, Liu D, Ma S, Dai Y, Zhang X (2020). Identification and expression of the multidrug and toxic compound extrusion (*MATE*) gene family in *Capsicum annuum* and *Solanum tuberosum*. Plants..

[56] Li XF, Zuo FH, Ling GZ, Li YY, Yu YX, Yang PQ (2009). Secretion of citrate from roots in response to aluminum and low phosphorus stresses in *Stylosanthes*. Plant Soil.

[57] Haran S, Logendra S, Seskar M, Bratanova M, Raskin I (2000). Characterization of Arabidopsis acid phosphatase promoter and regulation of acid phosphatase expression. Plant Physiol.

[58] Zhang Q, Wang C, Tian J, Li K, Shou H (2011). Identification of rice purple acid phosphatases related to phosphate starvation signalling. Plant Biol.

[59] Li C, Gui S, Yang T, Walk T, Wang X, Liao H (2012). Identification of soybean purple acid phosphatase genes and their expression responses to phosphorus availability and symbiosis. Ann Bot.

[60] Luo J, Liu Y, Zhang H, Wang J, Chen Z, Luo L (2020). Metabolic alterations provide insights into *Stylosanthes* roots responding to phosphorus deficiency. BMC Plant Biol.

[61] Valdés-López O, Arenas-Huertero C, Ramírez M, Girard L, Sánchez F, Vance CP (2008). Essential role of MYB transcription factor: PvPHR1 and microRNA: PvmiR399 in phosphorus-deficiency signalling in common bean roots. Plant Cell Environ.

[62] Guo M, Ruan W, Li C, Huang F, Zeng M, Liu Y (2015). Integrative comparison of the role of the PHOSPHATE RESPONSE1 subfamily in phosphate signaling and homeostasis in rice. Plant Physiol.

[63] Sun L, Song L, Zhang Y, Zheng Z, Liu D (2016). Arabidopsis PHL2 and PHR1 act redundantly as the key components of the central regulatory system controlling transcriptional responses to phosphate starvation. Plant Physiol.

[64] Xue YB, Xiao BX, Zhu SN, Mo XH, Liang CY, Tian J (2017). *GmPHR25*, a *GmPHR* member up-regulated by phosphate starvation, controls phosphate homeostasis in soybean. J Exp Bot.

[65] Chen YF, Li LQ, Xu Q, Kong YH, Wang H, Wu WH (2009). The WRKY6 transcription factor modulates *PHOSPHATE1* expression in response to low Pi stress in Arabidopsis. Plant Cell.

[66] Devaiah BN, Karthikeyan AS, Raghothama KG (2007). WRKY75 transcription factor is a modulator of phosphate acquisition and root development in Arabidopsis. Plant Physiol.

[67] Yao Z, Tian J, Liao H (2014). Comparative characterization of *GmSPX* members reveals that *GmSPX3* is involved in phosphate homeostasis in soybean. Ann Bot.

[68] Duan K, Yi K, Dang L, Huang H, Wu W, Wu P (2008). Characterization of a sub-family of Arabidopsis genes with the SPX domain reveals their diverse functions in plant tolerance to phosphorus starvation. Plant J.

[69] Puga MI, Mateos I, Charukesi R, Wang Z, Franco-Zorrilla JM, de Lorenzo L (2014). SPX1 is a phosphate-dependent inhibitor of PHOSPHATEF STARVATION RESPONSE 1 in *Arabidopsis*. Proc Natl Acad Sci U S A.

[70] Lv Q, Zhong Y, Wang Y, Wang Z, Zhang L, Shi J (2014). SPX4 negatively regulates phosphate signaling and homeostasis through its interaction with PHR2 in rice. Plant Cell.

[71] Jia Y, Li X, Liu Q, Hu X, Li J, Dong R (2020). Physiological and transcriptomic analyses reveal the roles of secondary metabolism in the adaptive responses of *Stylosanthes* to manganese toxicity. BMC Genomics.

[72] Murphy J, Riley JP (1962). A modified single solution method for the determination of phosphate in natural waters. Anal Chim Acta.

[73] Bradford MM (1976). A rapid and sensitive method for the quantitation of microgram quantities of protein utilizing the principle of protein-dye binding. Anal Biochem.

[74] Li B, Dewey CN (2011). RSEM: accurate transcript quantification from RNA-Seq data with or without a reference genome. BMC Bioinformatics.

[75] Love MI, Huber W, Anders S (2014). Moderated estimation of fold change and dispersion for RNA-seq data with DESeq2. Genome Biol.

[76] Kanehisa M, Goto S (2000). KEGG: Kyoto encyclopedia of genes and genomes. Nucleic Acids Res.

