# Supplementary material for: Physiological responses and transcriptomic changes reveal the mechanisms underlying adaptation of Stylosanthes guianensis to phosphorus deficiency
Source: BMC Plant Biol. 2021 Oct 13;21:466. doi: 10.1186/s12870-021-03249-2 (PMC8513372; doi:10.1186/s12870-021-03249-2)
Supplement: Supplementary file 10 — Additional file 10: Figure S2. Heatmap analysis of the DEGs belonging to SPX containing proteins. The transcripts of DEGs were normalized as log2(FPKM+1). Gene IDs were showed by the legend on the right. Expression levels ranged from red to blue indicate high to low expression for genes, respectively. LP and HP represent 0 and 250 μM KH2PO4 supply treatments, respectively. [file 12870_2021_3249_MOESM10_ESM.pdf]

Fig. S2

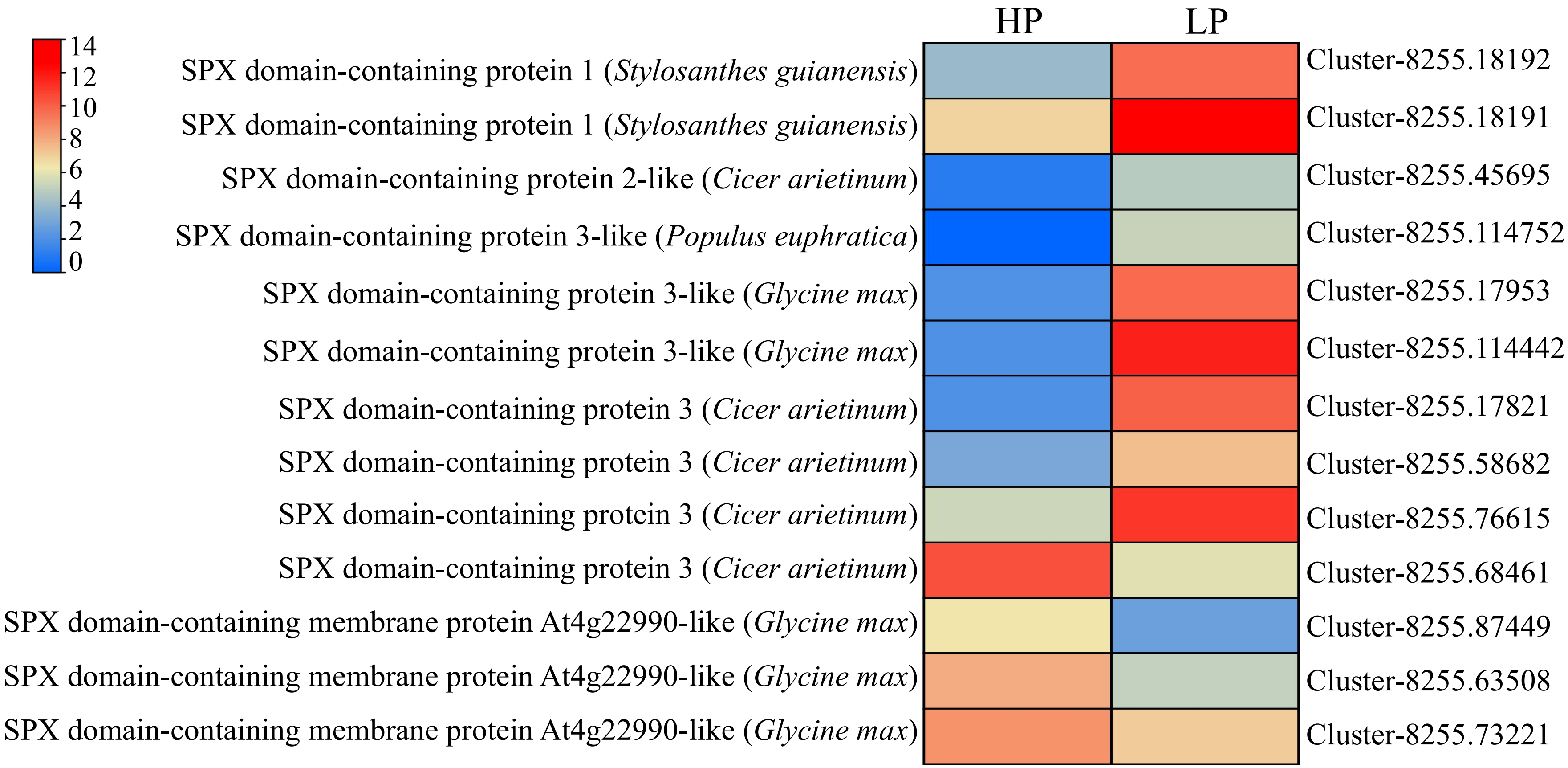

Fig. S2. Heatmap analysis of the DEGs belonging to SPX containing proteins. The transcripts of DEGs were normalized as log2(FPKM+1). Gene IDs were showed by the legend on the right. Expression levels ranged from red to blue indicate high to low expression for genes, respectively. LP and HP represent 0 and 250 μM KH_2PO_4 supply treatments, respectively.
