# Supplementary material for: Physiological responses and transcriptomic changes reveal the mechanisms underlying adaptation of Stylosanthes guianensis to phosphorus deficiency
Source: BMC Plant Biol. 2021 Oct 13;21:466. doi: 10.1186/s12870-021-03249-2 (PMC8513372; doi:10.1186/s12870-021-03249-2)
Supplement: Supplementary file 11 — Additional file 11: Figure S3. Correlation analysis of gene expression between transcriptome data and qRT-PCR results. Nine up-regulated and four down-regulated DEGs were selected for qRT-PCR analysis. Transcriptome data were plotted against data from qRT-PCR. Data are presented on a log2 scale. [file 12870_2021_3249_MOESM11_ESM.pdf]

Fig. S3

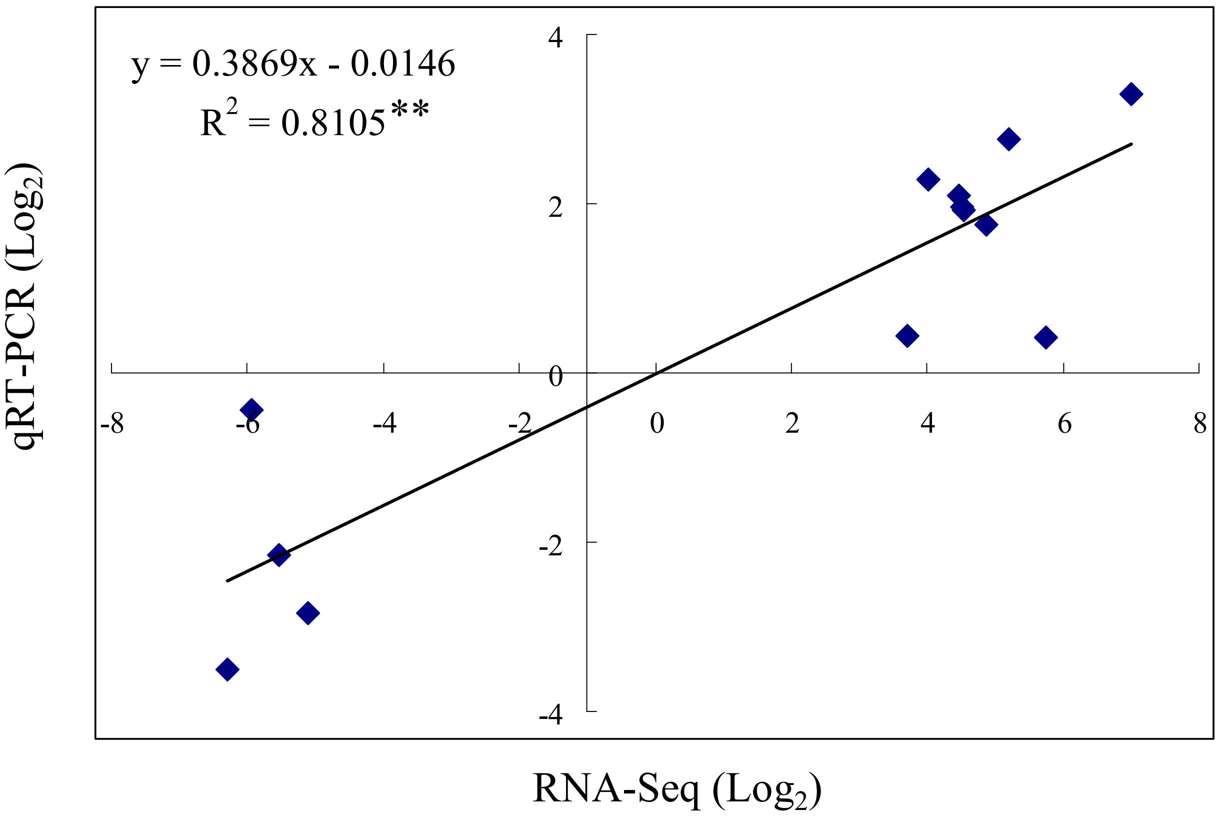

Fig. S3. Correlation analysis of gene expression between transcriptome data and qRT-PCR results. Nine up-regulated and four down-regulated DEGs were selected for qRT-PCR analysis. Transcriptome data were plotted against data from qRT-PCR. Data are presented on a $\log_2$ scale.
